# Effects of Electronegativity and Hydration Energy on the Selective Adsorption of Heavy Metal Ions by Synthetic NaX Zeolite

**DOI:** 10.3390/ma14154066

**Published:** 2021-07-21

**Authors:** Xianyuan Fan, Hong Liu, Emmanuella Anang, Dajun Ren

**Affiliations:** 1 Department of Resource and Environmental Engineering, Wuhan University of Science and Technology, Wuhan 430081, China; fanxianyuan@wust.edu.cn (X.F.); anangemmanuella@gmail.com (E.A.); rendajun@wust.edu.cn (D.R.); 2 Hubei Key Laboratory for Efficient Utilization and Agglomeration of Metallurgic Mineral Resources, Wuhan University of Science and Technology, Wuhan 430081, China

**Keywords:** NaX zeolite, selective adsorption, heavy metal ion, electronegativity, hydration energy

## Abstract

The adsorption capacity of synthetic NaX zeolite for Pb^2+^, Cd^2+^, Cu^2+^ and Zn^2+^ in single and multi-component systems were investigated. The effects of electronegativity and hydration energy on the selective adsorption, as well as potential selective adsorption mechanism of the NaX zeolite for Pb^2+^, Cd^2+^, Cu^2+^ and Zn^2+^ were also discussed. The maximum adsorption capacity order of the heavy metals in the single system was Pb^2+^ > Cd^2+^ > Cu^2+^ > Zn^2+^, and this could be related to their hydration energy and electronegativity. The values of the separation factors (α) and affinity constant (K_EL_) in different binary systems indicated that Pb^2+^ was preferentially adsorbed, and Zn^2+^ presented the lowest affinity for NaX zeolite. The selective adsorption capacities of the metals were in the order, Pb^2+^ > Cd^2+^ ≈ Cu^2+^ > Zn^2+^. The trend for the selective adsorption of NaX zeolite in ternary and quaternary systems was consistent with that in the binary systems. Pb^2+^ and Cu^2+^ reduced the stability of the Si-O-Al bonds and the double six-membered rings in the NaX framework, due to the high electronegativity of Pb^2+^ and Cu^2+^ than that of Al^3+^. The selective adsorption mechanism of NaX zeolite for the high electronegative metal ions could mainly result from the negatively charged O in the Si-O-Al structure of the NaX zeolite, hence heavy metal ions with high electronegativity display a strong affinity for the electron cloud of the oxygen atoms in the Si-O-Al. This study could evaluate the application and efficiency of zeolite in separating and recovering certain metal ions from industrial wastewater.

## 1. Introduction

Heavy metal ions such as Pb(II), Cr(VI), Cu(II), Zn(II), Hg(II) and Ni(II), are highly toxic, carcinogenic, difficult to degrade, and accumulate in living organisms when they get into the food chain [1]. Their presence in the ecosystem pose great danger to both human and environmental health, thus, the removal of such toxic substances is of much interest to researchers. Many conventional and modern methods aimed at removing heavy metals from environmental media have been developed and adopted over the years. Among the various techniques for heavy metal removal in wastewater, such as chemical precipitation, chemical reduction, ion-exchange, electrochemical treatment, reverse osmosis and membrane technologies, adsorption is regarded as the simplest, most effective and environmentally friendly remediation process [2,3].

Adsorption which denotes the attachment of a substance onto a surface could represent a feasible solution to ion exchange, and removal of heavy metals by natural or synthetic substrates [4]. Adsorbents, which may include clay, activated carbon, hydroxyapatite, resin or chitosan, are essential components in the adsorption process, and are expected to have high adsorption interactions with target contaminants in order to effectively remove them from wastewater [3,5,6]. Clay minerals, such as bentonite, zeolite and montmorillonite present several advantages including large specific surface areas, large ion-exchange capacities for heavy metal ions and widespread deposits [3,7,8,9]. However, selectivity for heavy metal ions, adsorption capacities and stability are relatively low in natural clay minerals, particularly, zeolites. In contrast, the adsorption capacities of synthetic zeolites are higher than those of natural zeolites due to their larger specific surface areas, controlled structural compositions, high thermal and chemical stability [10,11,12]. Therefore, synthetic zeolites display great potential for the treatment of wastewater containing heavy metals. 

Zeolite X is a three-dimensional crystal structure that is rich in synthetic Al. Its building blocks are made up of sodalite cells that are tetrahedrally interconnected at single six rings by bridging oxygen atoms to result in double six rings [13]. Application and investigation of various synthetic zeolites to remove heavy metals from wastewater have been mainly focused on the adsorption of single heavy metal ions [11,14,15,16,17], with few studies that are considering selective ion adsorption in multi-component systems [18,19]. Industrial wastewater usually contains two or more metals, and the presence of coexisting ions in the solution will inevitably affect the adsorption capacity of the materials to target pollutants. When ions are present in binary or multiple aqueous solutions with regards to the combined impact of the different heavy metal ions and synthetic zeolites, the conclusion of selective adsorption between metals species is not quite consistent [20,21]. 

The [AlO_4_] tetrahedral units in the synthetic zeolites’ frameworks are negatively charged, and could be neutralized by alkali metal cations (Na^+^, Ca^2+^, K^+^) introduced during the synthetic process. The alkali metal cations can easily be exchanged because they are free within the zeolite frameworks [22,23,24,25]. The pore size of synthetic zeolites may be an important factor affecting their selective adsorption, particularly when the diameter of hydrated ions is larger, metal ions may be excluded. Dehydration facilitates the movement of ions into the zeolite channels [26,27]. Therefore, the selective adsorption of synthetic zeolites for heavy metal ions may be related to the diameter and hydration energy of the corresponding hydrated ion.

A few studies [10,28,29,30] reported that heavy metal cations with low electronegativity can be easily replaced in aluminosilicate-based materials by others with higher electronegativity. For example, Xu et al. [29] found that Pb^2+^ was adsorbed on synthetic cancrinite by replacing Cd^2+^, due to its high electronegativity, as compared to Cd^2+^. According to Chao et al. [30], both K^+^ and H^+^ were replaced by Pb^2+^ in Si-O-K and Si-O-H type aluminosilicates due to the high electronegativity of Pb^2+^, but the metal uptake capacity was the same. Therefore, the electronegativity of heavy metals may also affect the selective adsorption behavior of aluminosilicate-based materials. Research on this phenomenon is rather limited. 

The selective adsorption of different heavy metals on synthetic zeolites and the underlying mechanisms are essential for the proper application of synthetic zeolites in the removal of heavy metal ions. In the present study, NaX zeolites with high ion-exchange capacity were selected as model materials. Common pollutants present in industrial wastewater such as Pb^2+^, Cd^2+^, Cu^2+^ and Zn^2+^ were also selected. The adsorption performances of NaX zeolites for Pb^2+^, Cd^2+^, Cu^2+^ and Zn^2+^ in single and multi-component solutions were systematically examined. The effects of electronegativity and hydration energy of Pb^2+^, Cd^2+^, Cu^2+^ and Zn^2+^ on the selective adsorption of NaX zeolites were investigated. Furthermore, structural properties of NaX zeolites were considered in order to determine the relationship between the mechanisms of selective adsorption with hydration energy and electronegativity.

## 2. Materials and Methods

### 2.1. Materials

The NaX zeolite used in the present research was supplied by Nankai University Catalyst Co. Ltd. (Tianjin, China). All reagents, including cupric nitrate, lead nitrate, cadmium nitrate, zinc nitrate, sodium hydroxide, and nitric acid were of analytical reagent grade and purchased from Sinopharm Chemical Reagent Co. (Shanghai, China). The pH of the tested solution was adjusted to the desired value by adding diluted sodium hydroxide or nitric acid. 

### 2.2. Adsorption Experiments

#### 2.2.1. Adsorption Isotherms

Adsorption equilibrium isotherms for single and multi-component systems containing Pb^2+^, Cd^2+^, Cu^2+^, and Zn^2+^ were obtained at an optimum pH of 5 and temperature, 298 K. Batch adsorption experiments were carried out in a 1.0 L beaker. A volume of 0.8 L of a single or multi metal solution and 0.80 g of NaX zeolite were placed in the beaker. Later, the mixture was stirred for 24 h using a rotating rate of 160 rpm until equilibrium was reached. In mono-component adsorption systems, the initial concentrations for Pb^2+^, Cd^2+^, Cu^2+^ and Zn^2+^ varied between 0.5 mmol/L and 5.0 mmol/L. In binary solutions, the target metal ion concentrations varied over the range of 1.0 to 4.0 mmol/L. In addition, concentrations of the interfering metal species were 1.0, 2.0, 3.0, and 4.0 mmol/L, respectively. In ternary and quaternary solutions, the equimolar concentrations of each heavy metal ion were 0.25, 0.5, 1.0, 2.0, 3.0 and 4.0 mmol/L. The concentrations of the metal ions in the residual aqueous phase were analyzed with a flame atomic absorption spectrophotometer (novAA350, Analytikjena, Germany). The amount of metal adsorbed per unit mass of NaX zeolite was determined using Equation (1),

(1) $$Q_{e}=\frac{V\left(C_{0}{-C}_{e}\right)}{m}$$

where C_0_ and C_e_ are the initial and equilibrium metal concentrations (mmol/L), respectively; m is the mass of NaX (g); V is the volume of the solution (L); and Q_e_ is the equilibrium adsorption capacity (mmol/g).

The concentrations of Na^+^ in the residual aqueous phase of single systems were analyzed with an ion chromatograph (883, Vantone, Switzerland). The concentration of Na^+^ in 1.0 g/L NaX aqueous solution was taken as blank background.

#### 2.2.2. Time Profiles of Competitive Adsorption Process in the Binary Systems

Solutions containing single or binary ions at concentrations, 2.0 and 3.0 mmol/L, were mixed with NaX zeolites (solid concentration 1.0 g/L, total volume 0.8 L). In binary systems, a molar ratio of 1:1 was selected. Mixtures were adjusted at an initial pH of 5 and mechanically stirred at 160 rpm at a temperature of 298 K. 0.005 L aliquots were taken at the pre-settled time intervals and concentrations of the adsorbed ions were calculated using Equation (1).

### 2.3. Modeling of Metal Adsorption

Adsorption isotherms in single systems were obtained using Langmuir and Freundlich models as presented in Equations (2) and (3),

(2) $$Q_{e}=\frac{K_{L}Q_{m}C_{e}}{{1+K}_{L}C_{e}}$$

(3) $$Q_{e}{=K}_{f}C_{e}^{1/n}$$

where C_e_ is the equilibrium concentration (mmol/L); Q_e_ is the equilibrium adsorption capacity (mmol/g); K_L_ is the affinity parameter or Langmuir sorption constant (L/mmol), which reflects the sorption free energy; Q_m_ is the capacity parameter (mmol/g); K_f_ and n are the Freundlich constant isotherm parameters.

In binary systems, the selectivity of competitive adsorption was concentration dependent [31]. The separation factor (an index of selectivity) is expressed in Equation (4):

(4) $$\alpha_{2}^{1}=\frac{Q_{e1}{\text{×}C}_{e2}}{Q_{e2}{\text{×}C}_{e1}}$$

In order to evaluate the affinity of NaX zeolite towards the heavy metal ions, the adsorption data of binary systems were simulated with the extended Langmuir model [32,33]. The extended Langmuir model is expressed as [8,31,34]:

(5) $$Q_{e,i}=\frac{Q_{\max}K_{\mathrm{EL},i}C_{e,i}}{{1+K}_{\mathrm{EL},i}C_{e,i}{+K}_{\mathrm{EL},j}C_{e,j}}$$

where Q_max_ is the maximum capacity (mmol/g) and K_EL,i_ is the affinity parameter or Langmuir adsorption constant (L/mmol). 

The three-dimensional isotherm surfaces were generated using MATLAB 2014b, with a minimum value of the residual sum of squares (RSS) and the chi-squared (χ^2^) which could be defined as:

(6) $$\mathrm{RSS}=\sum {\left(Q_{e,\exp}{-Q}_{e,\mathrm{fit}}\right)}^{2}$$

(7) $$\chi^{2}=\sum \frac{{\left(Q_{e,\exp}{-Q}_{e,\mathrm{fit}}\right)}^{2}}{Q_{e,\mathrm{fit}}}$$

### 2.4. Characterization 

Fourier transform infrared spectrometer (Vertex70, Bruker, Germany) was used to analyze functional groups present in the NaX zeolites loaded with heavy metal ions. The specific surface areas and pore parameters of materials were determined using N_2_ adsorption-desorption method by a specific surface area analyzer (Micromeritics ASAP 2020, Atlanta, GA, USA). The X-ray diffraction (XRD) measurements for NaX zeolites powders were conducted by an X-ray diffractometer (max-IIIA, Rigaku D, Japan). The morphologies of NaX zeolites were characterized by a scanning electron microscopy (SEM) (Nova 400 Nano, FEI, Hillsboro, OR, USA). The laser particle size analyzer (Mastersizer 2000, Melvin, UK) was used to determine particle size distribution

## 3. Results and Discussion

### 3.1. Characterizations of NaX Zeolites

The elemental content of commercial NaX zeolite and the mole of Si/Al are shown in Table 1. The mole of Si/Al was 1.35, and the exchangeable cations were mostly Na^+^. Based on the contents of K, Na, Mg and Ca, the divalent cation exchange capacity (CEC) was calculated to be 2.393 mmol/g in NaX zeolite.

**Figure 1:**
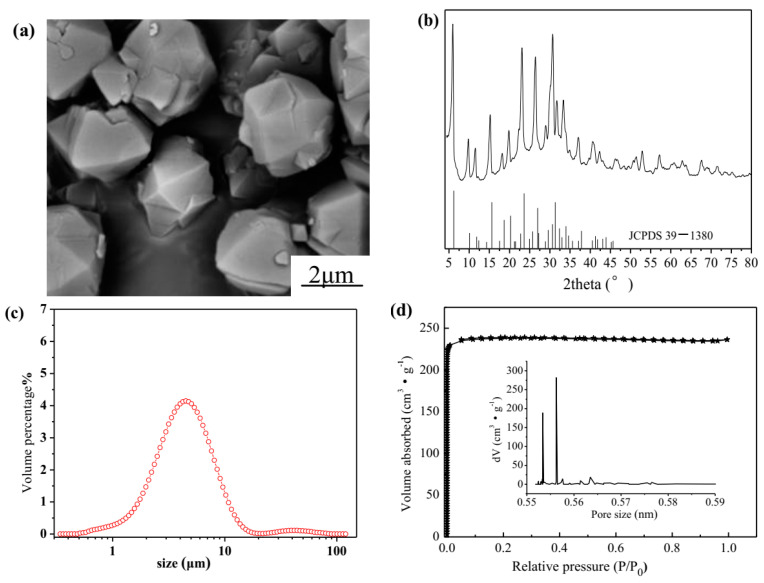
Characterization of NaX zeolite, SEM image (**a**), XRD pattern (**b**), particle size distribution (**c**), N_2_ adsorption-desorption isotherms and Pore size distribution (inset) (**d**).

The SEM image, XRD pattern, particle size distribution, N_2_ adsorption-desorption isotherms and pore size distribution (inset) of NaX zeolite are shown in Figure 1. The commercial NaX zeolite exhibits a cubic crystal system of octahedron (Figure 1a), and particle size distribution d (0.5) of 4.213 μm (Figure 1c). It was found that NaX zeolite exhibits a narrow pore size distribution of 0.55–0.56 nm (Figure 1d). The BET specific surface area was 712.7 m^2^·g^−1^, and the shape of N_2_ adsorption-desorption isotherms was typically characteristic of microporous materials.

### 3.2. Adsorption of Pb^2+^, Cd^2+^, Cu^2+^ and Zn^2+^ by NaX Zeolites in a Single System 

**Figure 2:**
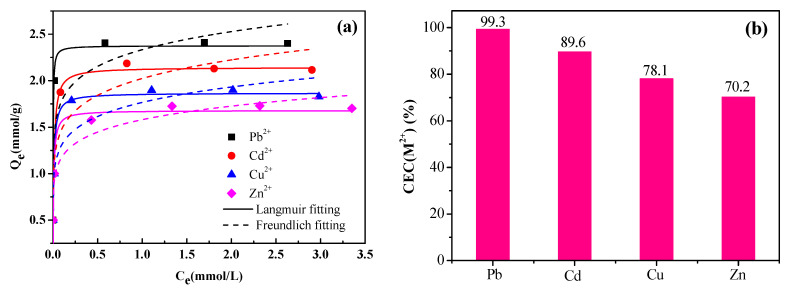

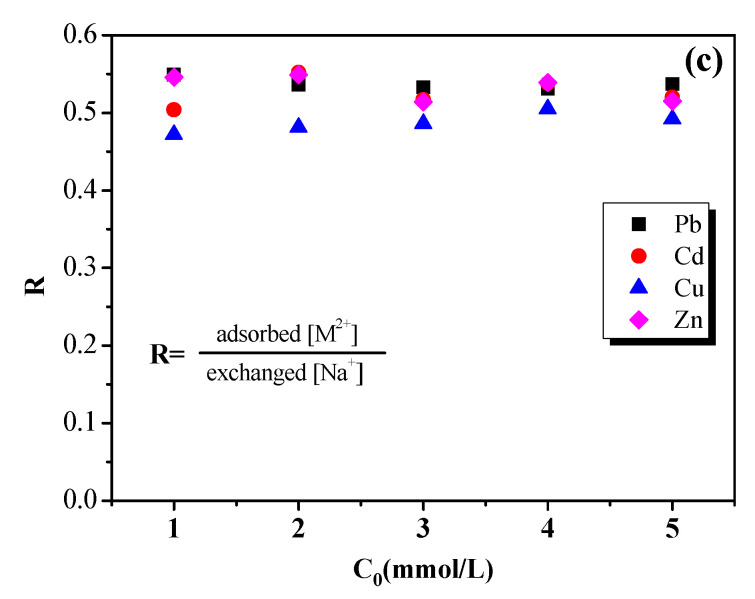
(**a**) NaX adsorption isotherms for Pb^2+^, Cd^2+^, Cu^2+^ and Zn^2+^; (**b**) the M^2+^ amounts adsorbed as % of the CEC; and (**c**) the ratio (R) of adsorbed [M^2+^] to exchanged [Na^+^] during the adsorption process.

In order to evaluate the adsorption capacity of NaX for different heavy metals, Pb^2+^, Cd^2+^, Cu^2+^ and Zn^2+^ were selected for adsorption experiments in a single system. The nonlinear isothermal fittings and fitting parameters are shown in Figure 2a and Table 2.

The results indicated that the Langmuir model justified the experimental data with an adequate correlation coefficient (R^2^ > 0.9), and the corresponding equilibrium adsorption capacity was 2.38, 2.14, 1.87 and 1.68 mmol/g for Pb^2+^, Cd^2+^, Cu^2+^ and Zn^2+^, respectively. The overall trend of adsorption capacity was in the order of Pb^2+^ > Cd^2+^ > Cu^2+^ > Zn^2+^.

The maximum adsorbed amounts of heavy metal ion (M^2+^) expressed as % of the divalent cation exchange capacity (CEC) of NaX zeolites were calculated using Equation (8), and the results are shown in Figure 2b. The results indicated that Pb^2+^ could exchange almost all exchangeable ions in NaX zeolites:

(8) $${\mathrm{CEC}(M}^{2+})\;(\%)=\frac{Q_{m}}{\mathrm{CEC}}\text{×}100\%.$$

The ratio of adsorbed heavy metal ion (M^2+^) to exchanged Na^+^ are shown in Figure 2c. The individual R values for the heavy metals were scattered around 0.5 (Figure 2c), thus indicating that the removal of heavy metal ions may be attributed to their exchange with Na^+^ ions present in the NaX matrix. Compared to the size of the supercage in NaX (0.74–0.78 nm), the hydration diameter of the heavy metals (Pb^2+^ (0.802 nm), Cd^2+^ (0.852 nm), Cu^2+^ (0.838 nm), and Zn^2+^ (0.860 nm)) are just a little large. Therefore, heavy metal ions were able to enter the supercage of NaX and exchange with Na^+^. Some of the hydration water was stripped from the solvated ions (dehydration). However, the exchange may rather be difficult because the cations are too big to diffuse through the 6-membered rings of the cubooctahedra and hexagonal prisms. The Pb^2+^ may lose all its hydration water to diffuse through the 6-membered rings (0.28 nm), but the others (Cd^2+^, Cu^2+^ or Zn^2+^) may not. Therefore Cd^2+^, Cu^2+^ or Zn^2+^ undergo partial exchange.

Dehydration depends on the hydration energy of the corresponding metal ions, because the lower the hydration energy, the easier it is for the metal ion to dehydrate. The order of the hydration energy for the heavy metal ions was Pb^2+^ (1502 KJ/mol) < Cd^2+^ (1828 KJ/mol) < Zn^2+^ (2058 KJ/mol) < Cu^2+^ (2121 KJ/mol) [35]. Pb^2+^ displayed the least hydration energy as compared to the other ions, thus hydrated Pb^2+^ can be easily dehydrated. Hence, NaX displayed more adsorption capacity for Pb^2+^, as compared to the rest of the heavy metal ions. Similarly, Cd^2+^ adsorption can be explained by the same rule. However, the adsorption capacity of Zn^2+^ and Cu^2+^ displayed cannot be due to the rule of hydration energy, while Selim [30] and Liu [36] obtained the similar phenomenon. This result could be related to Pauling electronegativity and hydrated ionic radius of Cu^2+^ and Zn^2+^ [30]. The hydration radius of hydrated Cu^2+^ is smaller than that of Zn^2+^. A larger hydrated radius means that the cationic center of charge is farther from the clay surface so the clay-cation electrostatic interaction is weaker [27]. Moreover, Pauling electronegativity of copper (1.9) is higher than that of zinc (1.65) [36]. Therefore, Cu^2+^ exhibited a greater affinity towards the NaX.

### 3.3. Selective Adsorption of Target Heavy Metals in Binary Systems by NaX Zeolites 

In order to investigate the effects of hydration energy and electronegativity on the selective adsorption of Pb^2+^, Cd^2+^, Cu^2+^ and Zn^2+^ using NaX, the following binary solution systems were designed: Cu^2+^-Pb^2+^, Cu^2+^-Cd^2+^, Cu^2+^-Zn^2+^ and Cd^2+^-Zn^2+^. The orders of hydration energy and electronegativity of the heavy metals in different binary systems are shown in Table 3. 

#### 3.3.1. Binary Adsorption Equilibrium Isotherm

**Figure 3:**
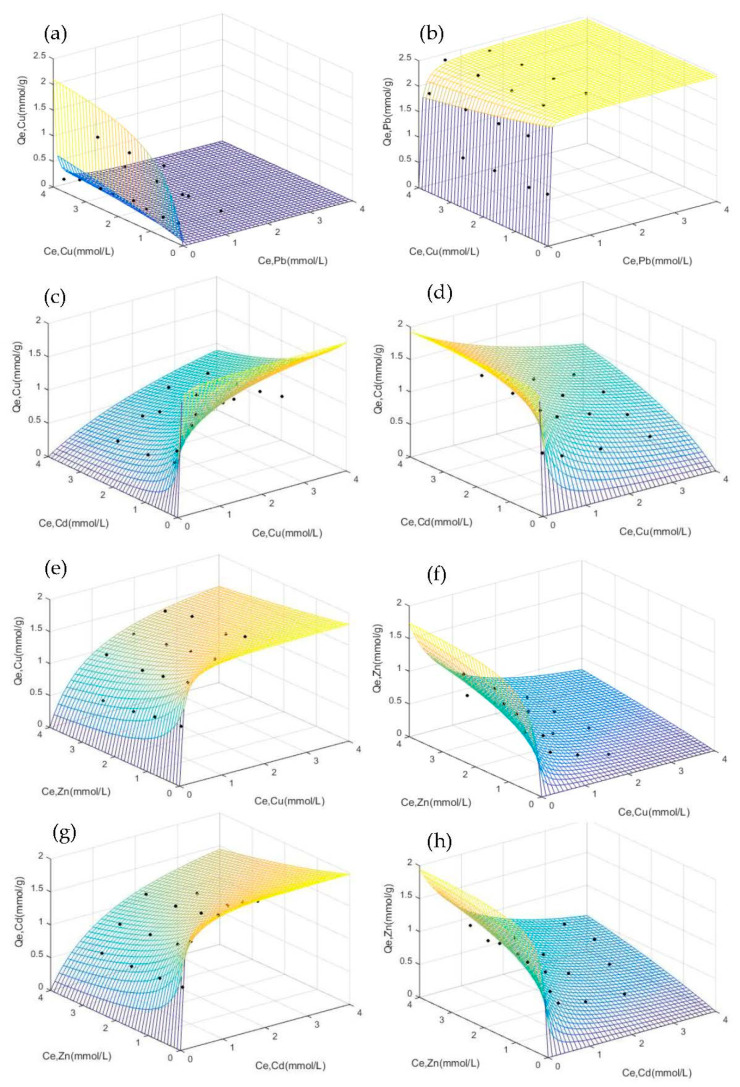
Adsorption capacity of heavy metals by NaX zeolite and three-dimensional isotherm surfaces simulated with the extended Langmuir isotherm model: Cu^2+^-Pb^2+^ binary system (**a**,**b**), Cu^2+^-Cd^2+^ binary system (**c**,**d**), Cu^2+^-Zn^2+^ binary system (**e**,**f**) and Cd^2+^-Zn^2+^ binary system (**g**,**h**).

Figure 3 shows adsorption isotherm and three-dimensional isotherm surfaces simulated with the extended Langmuir isothermal model for the adsorption capacity of each metal set present in the four binary systems. 

##### Cu^2+^-Pb^2+^ Binary System

In the Cu^2+^-Pb^2+^ binary system, Cu^2+^ served as the target ion, while Pb^2+^ was selected as the competitive ion during the process of Cu^2+^ adsorption. The Cu^2+^ adsorption surface isotherm displayed a concave downwards shape, but Pb^2+^ was closer to the XY plane particularly at low initial concentrations (Figure 3a). The adsorption capacity of Pb^2+^ gradually increased to 2.3~2.4 mmol/g with increasing initial Pb^2+^ concentration (Figure 3b). The maximum capacity of Pb^2+^ was almost equal to that of Pb^2+^ in a single solute (Figure 2). NaX will preferentially adsorb Pb^2+^, which displays lower hydration energy and higher electronegativity than Cu^2+^, and Cu^2+^ will be adsorbed only when the concentration of Pb^2+^ is insufficient for NaX to reach adsorption saturation. 

##### Cu^2+^-Cd^2+^ Binary System 

In the Cu^2+^-Cd^2+^ binary system, Cu^2+^ was selected as the target ion, while Cd^2+^ was selected as the competing ion. The adsorption capacity of Cu^2+^ increased with increasing Cu^2+^ concentrations but decreased when the Cd^2+^ concentration increased. The same effect was exhibited by the Cd^2+^ in the binary system (Figure 3c,d), but Cu^2+^ adsorption capacity gradually decreased to 0.92 mmol/g and Cd^2+^ adsorption capacity decreased to 0.93 mmol/g (when initial concentration C_0_,_Cd_ = 4.0 mmol/L and C_0,Cu_ = 4.0 mmol/L). It can be established that the NaX displayed similar selective adsorption behaviour with Cu^2+^ and Cd^2+^ even though the hydration energy of Cd^2+^ was smaller and more easily adsorbed, and the electronegativity of Cu^2+^ (1.9) was higher than Cd^2+^ (1.69). 

##### Cu^2+^-Zn^2+^ Binary System 

The adsorption capacity of Cu^2+^ (the target ion) was insignificantly affected by Zn^2+^ (the competing ion), thus, gradually increasing as Cu^2+^ concentration increased (Figure 3e) while Zn^2+^ decreased (Figure 3f). When the concentration of Cu^2+^ was set at 4.0 mmol/L and initial Zn^2+^ concentration increased, the adsorption capacity of Cu^2+^ decreased from 1.84 mmol/g to 1.41 mmol/g. The adsorption capacity of Zn^2+^ was only between 0.03 mmol/g and 0.26 mmol/g. NaX, thus, displayed better selective adsorption performance for Cu^2+^ which presents relatively high electronegativity and similar hydration energy with Zn^2+^ in the Cu^2+^-Zn^2+^ binary system.

##### Cd^2+^-Zn^2+^ Binary System

In the Cd^2+^-Zn^2+^ binary system, Zn^2+^ acted as the target ion, and Cd^2+^ as the competing ion. As shown in Figure 3g,h, the adsorption capacity of Zn^2+^ increased with increasing initial Zn^2+^ concentration. However, it gradually decreased until reaching a value of 0.80 mmol/g with increasing Cd^2+^ concentrations, and the adsorption capacity of Cd^2+^decreased to 1.40 mmol/g with increasing Zn^2+^ concentrations (when C_Cd_ = 4.0 mmol/L, C_0,Zn_ = 4.0 mmol/L). Since the electronegativity of Zn^2+^ (1.65) is almost similar to that of Cd^2+^ (1.69) and Cd^2+^ demonstrated easy adsorption by the NaX zeolite due to its small hydration energy, the selectivity of NaX zeolite to Cd^2+^ was greater than Zn^2+^. 

#### 3.3.2. Affinity Coefficient (K_EL_) and Separation Factor (α)

The simulated parameters of the extended Langmuir model, the maximum adsorption capacity Q_max_ and the affinity coefficient K_EL_ are shown in Table 4. The K_EL_ value indicates the affinity of the NaX zeolite towards the heavy metal ions. The K_Pb_ value in the Cu^2+^-Pb^2+^ binary system was almost 119 times that of the K_Cu_ value. The affinity of the NaX for Pb^2+^ in the binary system was therefore higher than that for Cu^2+^. Moreover, in the Cu^2+^-Cd^2+^, Cu^2+^-Zn^2+^ and Cd^2+^-Zn^2+^ systems, the K_EL_ ratio between the preferred ions (ions with high adsorption capacity) and the secondary species (ions with low adsorption capacity) were 1.15, 4.66, and 2.67 respectively. The affinity of NaX zeolite towards Cu^2+^ and Cd^2+^ was similar, and that for Cu^2+^ was greater than Zn^2+^. In addition, the NaX affinity for Cd^2+^ was slightly higher than Zn^2+^.

The separation factor, α, is an index of selectivity. The separation factors of the binary systems with equal molality ratio (1:1) obtained in this study are shown in Table 5. Selected initial concentrations (C_0_) of each heavy metal ion in the binary systems were 1.0, 2.0, 3.0 and 4.0 mmol/L. When C_0_ was 1.0 mmol/L, the $\alpha_{\mathrm{Pb}}^{\mathrm{Cu}}$, $\alpha_{\mathrm{Cd}}^{\mathrm{Cu}}$, $\alpha_{\mathrm{Zn}}^{\mathrm{Cu}}$ and $\alpha_{\mathrm{Zn}}^{\mathrm{Cd}}$ separation factors were approximately equal to 1, thus, the heavy metal ions in the binary system are all adsorbed when the NaX does not reach saturation. When C_0_ ≥ 2.0 mmol/L, the $\alpha_{\mathrm{Pb}}^{\mathrm{Cu}}$ value was smaller than 1, indicative of the good selectivity of NaX towards Pb^2+^; the $\alpha_{\mathrm{Cd}}^{\mathrm{Cu}}$ value was close to 1, indicating that the selectivity of NaX towards Cu^2+^ and Cd^2+^ was almost the same; the $\alpha_{\mathrm{Zn}}^{\mathrm{Cu}}$ and $\alpha_{\mathrm{Zn}}^{\mathrm{Cd}}$ values were greater than 1, indicating that NaX preferred Cu^2+^ and Cd^2+^ over Zn^2+^.

#### 3.3.3. Process of the Selective Adsorption in the Binary Systems 

**Figure 4:**
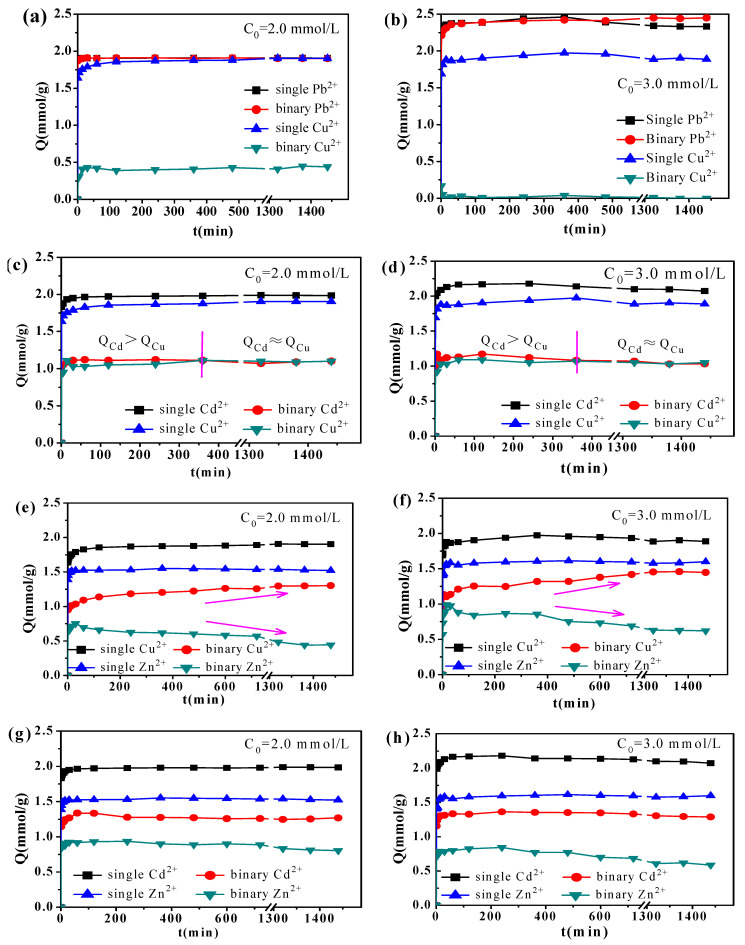
Time profiles of heavy metals adsorption in binary systems using NaX at equal molar ratio: Cu^2+^-Pb^2+^ binary system (**a**,**b**), Cu^2+^-Cd^2+^ binary system (**c**,**d**), Cu^2+^-Zn^2+^ binary system (**e**,**f**), and Cd^2+^-Zn^2+^ binary system (**g**,**h**).

The process of the selective adsorption for the Cu^2+^-Pb^2+^, Cu^2+^-Cd^2+^, Cu^2+^-Zn^2+^ and Cd^2+^-Zn^2+^ binary systems under equimolar concentrations are shown in Figure 4. The adsorption capacity for Pb^2+^ in the binary system was consistent with that in the single system, but the adsorption capacity for Cu^2+^ significantly reduced in the binary system. This was evident under both 2.0 and 3.0 mmol/L initial concentrations (Figure 4a,b). Cu^2+^ adsorbed by the NaX was replaced by Pb^2+^ within 10 min when the Pb^2+^ amount superceded the adsorption capacity of NaX (Figure 4b). Cu^2+^ and Cd^2+^ demonstrated low adsorption capacity in the binary system under both 2.0 mmol/L and 3.0 mmol/L initial concentrations, but higher adsorption capacity in the single system (Figure 4c,d). Adsorbed Cd^2+^ in the binary system was gradually replaced by Cu^2+^, which displays higher electronegativity. At the end of the process, the amounts of Cu^2+^ and Cd^2+^ adsorbed by the NaX were equivalent. In the Cu^2+^-Zn^2+^ binary system, Zn^2+^ adsorbed was gradually replaced by Cu^2+^, which is more electronegative than Zn^2+^ (Figure 4e,f). The adsorption capacity of Zn^2+^ by the NaX varied significantly, particularly in the 3 mmol/L initial concentration in both single and binary systems (Figure 4f). Zn^2+^ slowly decreased with increasing reaction time, while the adsorption capacity for Cd^2+^ did not show significant changes (Figure 4e,f). It is evident that Zn^2+^ and Cd^2+^ did not replace each other in the Cd^2+^-Zn^2+^ binary system.

Heavy metals that displayed small hydration energies were easier to dehydrate and subsequently be adsorbed by the NaX. However, in multiple ion systems, those ions with relatively high electronegativity were preferentially adsorbed by the NaX. Under 3.0 mmol/L initial concentration in the binary system, the molar masses of the ions exceeded the maximum adsorption capacity per gram of zeolite. The results of the selective adsorption by NaX indicated that: (i) Pb^2+^, which displayed low hydration energy and high electronegativity as compared to Cu^2+^ was preferentially adsorbed until reaching the saturation in the NaX, while Cu^2+^ was not adsorbed (Figure 4b); (ii) the adsorption capacity for Cu^2+^ with relatively high electronegativity was equivalent to that of Cd^2+^ which presented low hydration energy (Figure 4d); (iii) in the Cu^2+^-Zn^2+^ system, both elements presented similar hydration energies. Due to the higher electronegativity of Cu^2+^ as compared to Zn^2+^, the adsorption capacity for Cu^2+^ was more than three times that of Zn^2+^ (Figure 4f); (iv) Cd^2+^ and Zn^2+^ presented similar electronegativity values, while the adsorption capacity for Cd^2+^ which has low hydration energy, was only 1.8 times that of Zn^2+^ (Figure 4h). Moreover, the adsorption capacity for Zn^2+^ in the Cd^2+^-Zn^2+^ system was twice the one obtained for the Cu^2+^-Zn^2+^ system (Figure 4f,h). Therefore, adsorption sites on the NaX were more likely bound to heavy metal ions that displayed higher electronegativity. The selective adsorption capacities of the metals were in the order Pb^2+^ > Cd^2+^≈Cu^2+^ > Zn^2+^.

### 3.4. Selective Adsorption of Heavy Metal Ions by NaX in Ternary and Quaternary Systems 

**Figure 5:**
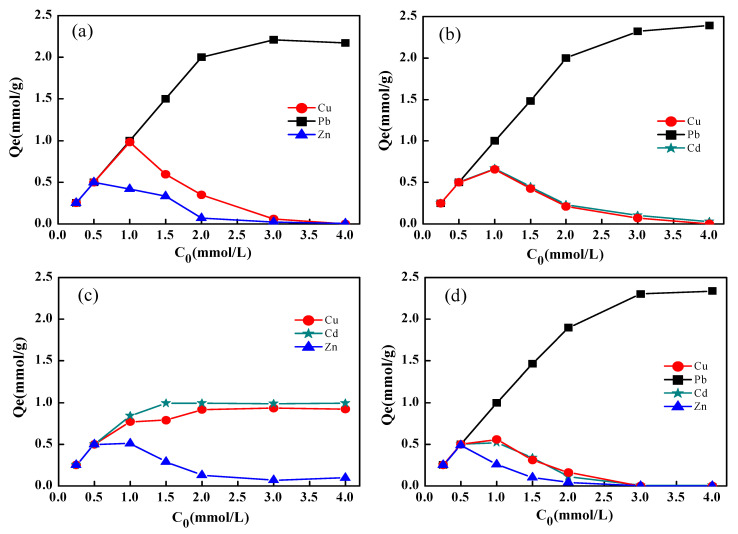
Adsorption capacity of NaX for heavy metal ions present in ternary and quaternary systems: (**a**) Pb^2+^-Cu^2+^-Zn^2+^, (**b**) Pb^2+^-Cd^2+^-Cu^2+^, (**c**) Cd^2+^-Cu^2+^-Zn^2+^ and (**d**) Pb^2+^-Cd^2+^-Cu^2+^-Zn^2+^.

The NaX adsorption capacities for Pb^2+^, Cd^2+^, Cu^2+^ or Zn^2+^ in the ternary (Pb^2+^-Cd^2+^-Zn^2+^, Pb^2+^-Cd^2+^-Cu^2+^ and Cd^2+^-Cu^2+^-Zn^2+^) and quaternary systems (Pb^2+^-Cd^2+^-Cu^2+^-Zn^2+^) at equimolar concentrations are shown in Figure 5. The results indicated that NaX was more selective towards Pb^2+^ than to the other three metals present in the multiple systems. Moreover, when the initial Pb^2+^ concentration was higher than 3.0 mmol/L, NaX did not adsorb other ions, hence, the adsorption capacity for Pb^2+^ was similar to that in the single system. The selective adsorption capacity of NaX for different heavy metal ions under the Pb^2+^-Cd^2+^-Zn^2+^, Pb^2+^-Cd^2+^-Cu^2+^, and Pb^2+^-Cd^2+^-Cu^2+^-Zn^2+^ systems was consistent with those observed in the binary systems (Figure 5a,b,d). However, in the Cd^2+^-Cu^2+^-Zn^2+^ group, when Cd^2+^ and Cu^2+^ concentrations were high enough to reach saturation in the NaX, Zn^2+^ adsorption was extremely low (Figure 5c).

### 3.5. Mechanisms of Selective Adsorption of Heavy Metal Ions by NaX 

**Figure 6:**
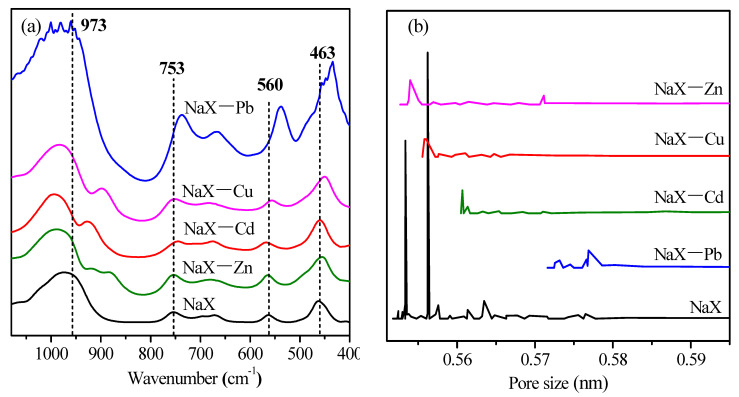
Infrared spectra (**a**) and pore size distribution (**b**) of NaX, NaX-Pb, NaX-Cd, NaX-Cu and NaX-Zn.

NaX-Pb, NaX-Cd, NaX-Cu and NaX-Zn represent NaX zeolite saturated with Pb^2+^, Cd^2+^, Cu^2+^ and Zn^2+^, respectively. The infrared spectra and pore size distribution of NaX, NaX-Pb, NaX-Cd, NaX-Cu and NaX-Zn zeolites are presented in Figure 6a,b. The BET specific surface area (S_BET_) and the pore parameters of NaX, NaX-Pb, NaX-Cd, NaX-Cu and NaX-Zn zeolites are presented in Table 6.

As shown in Figure 6a, the peak at 973 cm^−1^ in NaX was assigned to the asymmetric stretching vibration of Si-O-Si, Si-O-Al and O-Si-O groups, while the peak at 753 cm^−1^ corresponds to the symmetrical stretching vibration of the Si-O-Si or Si-O-Al groups [36]. The peak located at 463 cm^−1^ corresponds to bending vibrations of the Si-O-Si or Si-O-Al groups within the zeolite structure. Whereas, the peaks at 560 cm^−1^ and 671 cm^−1^ may be attributed to vibration modes of the double six-membered rings present in the zeolite framework [16,37].

NaX-Pb spectra showed that the peaks observed at 400–1000 cm^−1^ presented higher intensities as compared to those of the NaX. In addition, peaks at 753 cm^−1^, 560 cm^−1^ and 463 cm^−1^ displayed small shifts. However, according to the spectra, the chemical structure in the NaX-Cd and NaX-Zn remained almost unchanged. 

Structural changes of the NaX after adsorption can be explained by the electronegativity differences of heavy metal ions. The O in the Si-O-Al structure of the NaX was negatively charged. The greater the electronegativity of the heavy metal ions, the stronger the ability to attract the electron cloud of the oxygen atoms in the Si-O-Al. In NaX-Pb, Pb^2+^ displayed a stronger oxygen binding capacity given its higher electronegativity (2.33) as compared to those of Si (1.90) and Al (1.61). Therefore, Pb^2+^ reduced the stability of the Si-O-Al bonds and the double six-membered rings in the NaX framework. Similarly, the electronegativity of Cu (1.90), which was higher than that of Al (1.61), affected the stability of the Si-O-Al bonds within the NaX structure. The electronegativity of Cd (1.69) and Zn (1.65) were not bigger than that of Al (1.61), hence could not affect the structure of the NaX zeolite.

Figure 6b shows the pore size distribution of NaX, NaX-Pb, NaX-Cd, NaX-Cu and NaX-Zn. It can be found that the pore size of NaX-Pb, NaX-Cd, NaX-Cu and NaX-Zn are bigger than that of NaX. This is due to a divalent ion exchange with two Na^+^. Therefore, the position is empty, making the pore size larger. The position of the main peak in pore size distribution is NaX-Pb > NaX-Cd > NaX-Cu > NaX-Zn, which corresponds to the adsorption capacity of four kinds of heavy metal ions and the exchanged Na^+^ position. Zn^2+^ cannot enter the β-cage and hexagonal cylindrical cage with small pore size. The adsorption capacity of Zn^2+^ is the smallest, thus, the change in pore size of NaX zeolite is the least. The adsorption capacity of Pb^2+^ is the largest, and almost all the Na^+^ in NaX zeolite can be exchanged. Therefore, the pore size of NaX-Pb is the largest.

Compared with NaX, the t-plot micropore surface area and total pore volume of NaX-Pb, NaX-Cd, NaX-Cu, NaX-Zn reduced significantly (Table 6). For Pb and Cu with higher electronegativity, the pore volume and micropore surface area of NaX-Pb and NaX-Cu decreased more. The ions with high electronegativity were more likely to undergo acid-base coordination and hydrolysis, thus occupying pore volume.

**Figure 7:**
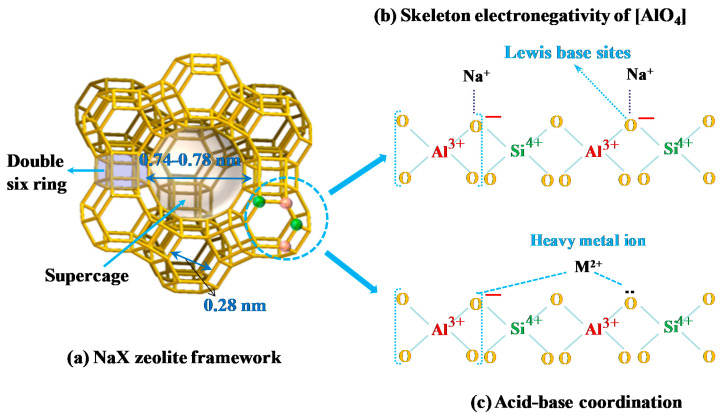
Diagram of the NaX zeolite framework (**a**), skeleton negatively charged of [AlO_4_] (**b**) and acid-base coordination (**c**).

The selectivity of NaX was related to the hydration energy and electronegativity of the adsorbed metal ions, which are relevant to the pore size and skeleton negative charge of the NaX. The supercage pore diameter of the NaX zeolite was 0.74–0.78 nm, and that of the 6-membered rings was 0.28 nm (Figure 7a), and the diameter of the hydrated heavy metal ions was larger than these values. Therefore, dehydrated heavy metal ions may enter the supercage pores and partially enter the 6-membered rings in the adsorption process. The oxygen skeleton present in the aluminized oxygen tetrahedron structure [AlO_4_] of the NaX which is also known as its structural base center is negatively charged (Figure 7b). According to the Lewis acid-base theory, heavy metal ions are acidic and are able to receive electrons [38]. Therefore, they can form acid-base coordinated structures with the O present in the base center of NaX (Figure 7c). The higher the electronegativity of the heavy metal ions, the stronger their ability to attract the electron clouds of negatively charged oxygen and, the stronger the coordinate bonds formed with O. Therefore, in multi-component systems, heavy metal ions with small hydration energy may enter the pores of NaX, but with the adsorption sites in the NaX that can be equally occupied by different heavy metal ions, their electronegativity is the key to determine their selective adsorption.

## 4. Conclusions

Due to the restricted interaction of the hydration diameter of the heavy metal ions and the pore size of the NaX, the maximum adsorption capacity in single system for Pb^2+^, Cd^2+^, Cu^2+^ and Zn^2+^ was 2.38 mmol/g, 2.14 mmol/g, 1.87 mmol/g, and 1.68 mmol/g, respectively. Heavy metal ions with relatively low hydration energy were easier to dehydrate before entering the NaX pore, and possessed higher adsorption capacity. The selective adsorption between two heavy metal ions resulted in an introduction of separation factor α and the affinity constant K_EL_ from the extended Langmuir isothermal model. The selective adsorption capacity and affinity followed the order of Pb^2+^ > Cd^2+^ ≈ Cu^2+^ > Zn^2+^, implying Pb^2+^ was the most favorable species and the affinity toward Zn^2+^ was the weakest. The separation factor achieved was about 1, implying the selectivity of NaX towards Cu^2+^ and Cd^2+^ was similar. Desorption of the metal ions with low electronegativity in binary solute was observed in the kinetic section and the possible mechanism could be described as direct displacement effect. The direct displacement mechanism of the adsorption process might prove that the electronegativity of metal ions should be a key factor associated with the selective adsorption on the NaX. The higher the electronegativity of the heavy metal ions, the stronger their ability to attract the electron clouds of negatively charged oxygen present in the NaX frameworks.

## Figures and Tables

**Table 1:** Element content of NaX zeolite (wt %).

Element	Si	Al	Ca	Mg	K	Na	n(Si/Al)
Mass percentage	18.18	12.94	0.07	0.07	0.04	10.65	1.35

**Table 2:** Isotherm parameters for Pb^2+^, Cd^2+^, Cu^2+^ and Zn^2+^ sorption using NaX.

Metal Ions	Langmuir			Freundlich		
	Q_m_ (mmol/g)	K_L_ (L/mmol)	R^2^	K_f_ (mmol/g)	n	R^2^
Pb^2+^	2.375	494.793	0.9881	2.3339	8.6640	0.7878
Cd^2+^	2.144	95.720	0.9625	2.0242	7.3697	0.7999
Cu^2+^	1.868	134.795	0.9390	1.7668	7.7328	0.8321
Zn^2+^	1.680	126.478	0.9017	1.5807	7.8530	0.9168

**Table 3:** The orders of hydration energy and electronegativity of the heavy metals in different binary systems.

Binary Systems	Hydration Energy	Electronegativity	Heavy Metals	Pauling Electronegativity	Hydration Energy (KJ/mol)
Cu^2+^-Pb^2+^	Cu^2+^ > Pb^2+^	Cu^2+^ < Pb^2+^	Pb^2+^	2.33	1502
Cu^2+^-Cd^2+^	Cu^2+^ > Cd^2+^	Cu^2+^ > Cd^2+^	Cd^2+^	1.69	1828
Cu^2+^-Zn^2+^	Cu^2+^ > Zn^2+^	Cu^2+^ > Zn^2+^	Cu^2+^	1.9	2121
Cd^2+^-Zn^2+^	Cd^2+^ < Zn^2+^	Cd^2+^ ≈ Zn^2+^	Zn^2+^	1.65	2058

**Table 4:** Multi-component isotherm model parameters for the adsorption of Cu^2+^, Pb^2+^, Zn^2+^ and Cd^2+^ at 303 K.

Binary Systems	Q_max_ (mmol/g)	K_EL_ (L/mmol)	RSS	χ^2^
Cu^2+^-Pb^2+^	2.416	K_Cu_ = 1.4391; K_Pb_ = 171.3544	2.3437	0.9701
Cu^2+^-Cd^2+^	1.932	K_Cu_ = 91.5320; K_Cd_ = 94.0259	1.6519	0.8550
Cu^2+^-Zn^2+^	1.851	K_Cu_ = 17.9153; K_Zn_ = 3.8476	0.9048	0.4888
Cd^2+^-Zn^2+^	2.004	K_Cd_ = 21.5285; K_Zn_ = 8.0486	3.1121	1.5529

**Table 5:** Separation factors (*α*) in binary systems at equal molar ratios.

C_0_ (mmol/L)	$\alpha_{\mathrm{Pb}}^{\mathrm{Cu}}$	$\alpha_{\mathrm{Cd}}^{\mathrm{Cu}}$	$\alpha_{\mathrm{Zn}}^{\mathrm{Cu}}$	$\alpha_{\mathrm{Zn}}^{\mathrm{Cd}}$
1.0	0.9802	1.0000	1.0242	0.9968
2.0	0.0077	0.9533	4.7668	1.9258
3.0	0.0027	0.9609	4.9634	2.5103
4.0	0.0089	0.9860	11.5534	1.6304

**Table 6:** The BET specific surface area (S_BET_) and pore parameters of NaX, NaX-Pb, NaX-Cd, NaX-Cu and NaX-Zn zeolites.

	Average Pore Size (nm)	S_BET_ (m^2^/g)	t-Plot Micropore Surface Area (m^2^/g)	Total Pore Volume (cm^3^/g)
NaX	0.556	712.7	701.9	0.3556
NaX-Pb	0.599	351.9	346.2	0.1716
NaX-Cd	0.611	551.3	545.8	0.2720
NaX-Cu	0.613	515.9	484.9	0.2467
NaX-Zn	0.611	592.2	578.8	0.2910

## Data Availability

All relevant data presented in the article are stored according to institutional requirements and as such are not available online. However, all data used in this manuscript can be made available upon request to the authors.
